# Dr. Beddoes's Notice of His Reports

**Published:** 1799-12

**Authors:** Thomas Beddoes

**Affiliations:** Rodney-Place, Clifton


### *To the Editors of the Medical and Phyſical Journal.*

Gentlemen,

As nobody, to my knowledge, has been moved by my exhortations, in *Conſiderations on Airs*, in the W. C. Contributions, and in my *Eſſay on Conſumption*, to try the effect of living with cows in phthiſis pulmonalis, I have lately, myſelf, put this practice to the teſt of experiment. I have gone to work on ſuch a ſcale, that I ſhall, in no long time, be able to ſtand before the public, and to ſay whether this expedient will produce any beneficial effect or not. I do not expect to jump all at once into a cure for the majority of caſes of true conſumption; but, by the analyſis, variation, and ſimplification of the method, I do certainly expect to diſcover ſomething valuable, at leaſt, in the way of relief.

Upon ſeeing one of my patients who had been ſubjected to the proceſs ceſs for about a month, an apothecary in Briſtol has thought it worth while to imitate it; and I hope others will alſo follow the example, now it can be done without the opprobrium of innovation.

Before your next Number appears, I ſhall, probably, publiſh the firſt part of my reports.

I am, GENTLEMEN,

Your moſt obedient ſervant,

THOMAS BEDDOES.

*Rodney-Place, Clifton,*

